# Supplementary material for: Genome-Wide Genetic Structure of Henan Indigenous Chicken Breeds
Source: Animals (Basel). 2023 Feb 19;13(4):753. doi: 10.3390/ani13040753 (PMC9952073; doi:10.3390/ani13040753)
Supplement: Supplementary file 1 [file animals-13-00753-s001.zip › animals-2191363- Supplementary Figures.pdf]

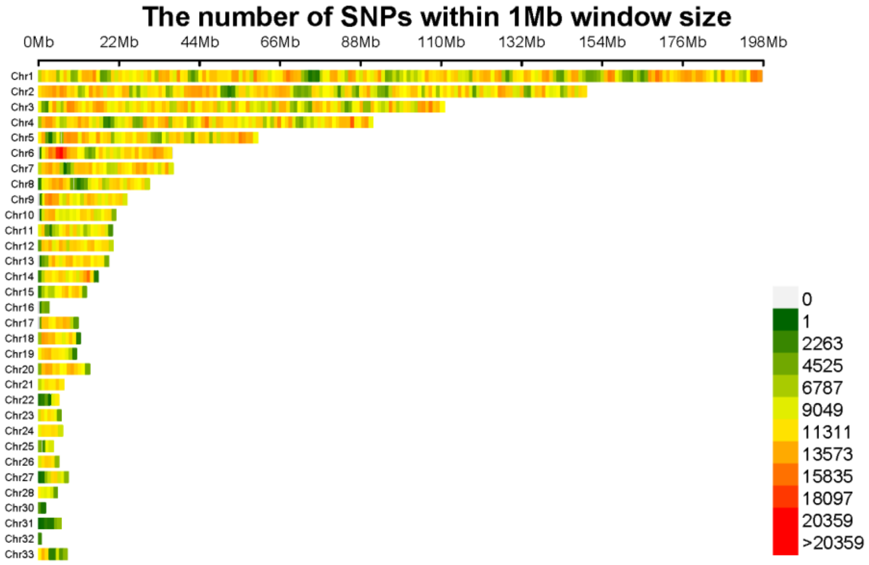

Figure S1: SNP density map.

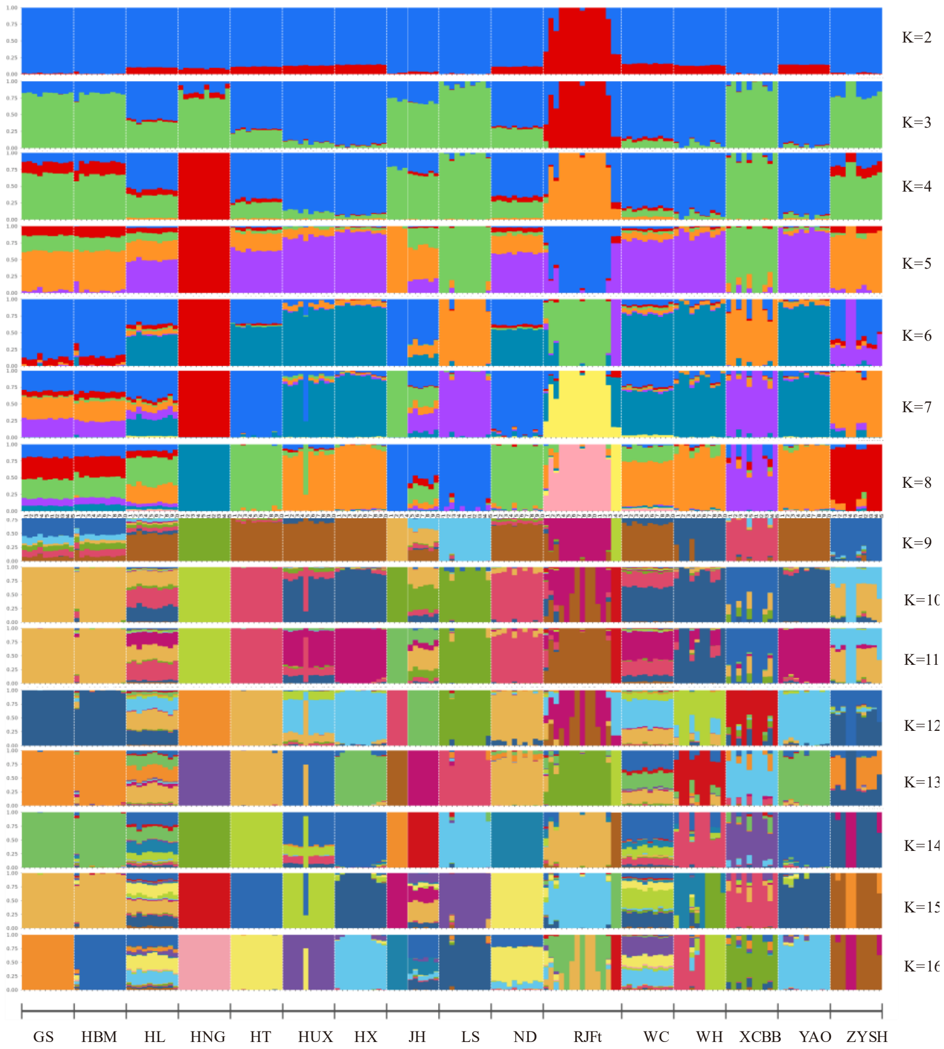

Figure S2. Admixture analysis across 16 chicken populations. Pro-portions of geneticancestry for 16 chicken populations with K=2-16 (K represents the number of inferred ancestral populations).

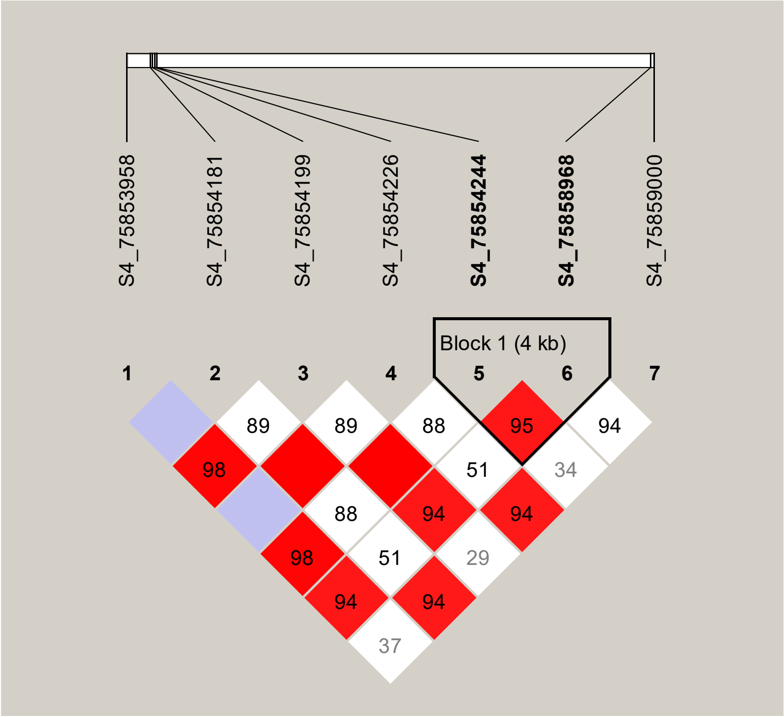

Figure S3: 6 SNPs on *LCORL* gene LD block.
